# Human cystic and alveolar echinococcosis in the Tibet Autonomous Region (TAR), China

**DOI:** 10.1017/S0022149X15000656

**Published:** 2015-08-14

**Authors:** X. Feng, X. Qi, L. Yang, X. Duan, B. Fang, Q. Gongsang, B. Bartholomot, D.A. Vuitton, H. Wen, P.S. Craig

**Affiliations:** 1 Xinjiang Hydatid Clinical Research Institute, The First Affiliated Hospital of Xinjiang Medical University, No.137 Liyushan South RD, Urumqi830054, Xinjiang, P. R. China; 2 Centre of Disease Prevention and Control, No. 21 Linkuo North RD, Lhasa, Tibet Autonomous Region; 3 WHO Collaborating Centre for Prevention and Treatment of Human Echinococcosis, SERF Research Unit EA 2276, University of Franche-Comte, 25030Besancon Cedex, France; 4 Cestode Zoonoses Research Group, School of Environment and Life Sciences, University of Salford, M5 4WT, UK

## Abstract

Human cystic echinococcosis (CE) is known to be endemic in the Tibet Autonomous Region (TAR), China; however, there is relatively little data from hospital records or community prevalence studies, and the situation regarding occurrence of human alveolar echinococcosis (AE) is unclear. Here we review the available reports about human echinococcosis in the seven prefectures of TAR. In addition, two pilot studies by mass screening using ultrasound (with serology) were undertaken (2006/7) in Dangxiong County of Lhasa Prefecture (north central TAR) and Dingqing County of Changdu Prefecture (eastern TAR). In Dangxiong County a prevalence of 9.9% (55/557) for human CE was obtained but no human AE cases were detected. By contrast, in Dingqing County (*N*= 232 persons screened), 11 CE cases (4.7%) and 12 AE cases (5.2%) (including one mixed CE and AE case) were diagnosed by ultrasound. Hospital records and published reports indicated that CE cases were recorded in all of seven prefectures in Tibet Autonomous Region, and AE cases in four prefectures. Incidence rates of human CE were estimated to range from 1.9 to 155 per 100,000 across the seven prefectures of TAR, with a regional incidence of 45.1 per 100,000. Incidence of AE was estimated to be between 0.6 and 2.8 cases per 100,000. Overall for TAR, human AE prevalence appeared relatively low; however, the pilot mass screening in Dingqing in eastern TAR indicated that human AE disease is a potential public health problem, possibly similar to that already well described in Tibetan communities bordering TAR in north-west Sichuan and south-west Qinghai provinces.

## Introduction

Human cystic echinococcosis (CE) and alveolar echinococcosis (AE) are helminthic zoonotic diseases caused by infection with the metacestode stages of *Echinococcus granulosus* (sensu lato) and *Echinococcus multilocularis*, respectively. Both are highly endemic in north-western China and are an important public health problem (Jiang, 2002; Craig 2004; Ministry of Health, 2005). A national echinococcosis control programme was implemented from 2006/7 in western China with emphasis on dog deworming, but has not yet been rolled out effectively in the more remote communities in western China, including north-west Xinjiang and much of the Tibet Autonomous Region (TAR) (Craig *et al.*, 2007, 2008; van Kesteren *et al.*, 2015). The burden of both human CE and AE disease is known to be particularly high in Tibetan communities located in north-west Sichuan and south-west Qinghai provinces on the eastern Tibetan plateau (Budke *et al.*, 2005; Li *et al.*, 2005, 2010).

The Tibet Autonomous Region covers 1.23 million km^2^ and is bordered to the north by Qinghai Province and Xinjiang Uygur Autonomous Region, to the east by Sichuan and Yunnan provinces, and to the south with Myanmar, India, Bhutan and Nepal. The TAR (capital Lhasa) forms a major part (47%) of the Qinghai–Tibetan Plateau (total area 2.5 million km^2^). With an average elevation over 4000 m and a population of 2.81 million, TAR has the lowest population density (2.3 inhabitants per km^2^) in China (National Bureau of Statistics of China, 2007). TAR is divided into one prefecture-level city (Lhasa) and six prefectures: Naqu (*Nagqu*), Ali (*Ngari*), Linzhi (*Nyinchi*), Changdu (*Qamdo*), Shannan and Rikaze (*Xigazê*). In addition, a significant Tibetan population is present in the high pasture regions of four other provinces of China (i.e. Qinghai, Sichuan, Gansu and Yunnan) with a total Tibetan population in China of approximately 6 million, including TAR.

Human cystic echinococcosis (CE) has been formally recognized as endemic in the TAR since 1987 (Hu *et al.*, 1987) and CE cases have been found continuously among the predominant nomadic pastoral population on the Tibetan plateau outside the TAR (in the neighbouring provinces of Sichuan, Qinghai and Gansu). Mass screening programmes for human echinococcosis, principally using portable ultrasound to detect abdominal CE or AE, have been undertaken in several Tibetan Autonomous Counties and Prefectures outside the TAR; particularly in Qinghai and Gansu provinces and in western Sichuan Province (Bai *et al.*, 2002; Schantz *et al.*, 2003; Li *et al.*, 2010). These studies revealed a very high prevalence of both CE and AE in Tibetan communities in Sichuan and Qinghai, with mean prevalences of 4–6% (although the prevalence at the township level ranges from 1 to 15%) for both CE and AE (Li *et al.*, 2010). In 2004, the Chinese Ministry of Health carried out a nationwide assessment of eight parasitic diseases, including echinococcosis (Ministry of Health, 2005). The highest overall ultrasound CE prevalence (2.4%) by ethnicity was in the Tibetan communities of TAR, north-west Sichuan and Qinghai Province. Despite these studies, there remains a relative lack of information regarding the prevalence and public health burden of echinococcosis (CE and AE) in the TAR.

In order to investigate the status of human echinococcosis in TAR, we reviewed published reports on echinococcosis in the TAR up to 2006/7, and also patient records from the People's Hospital of Changdu Prefecture in eastern TAR. In addition, we carried out pilot community studies based on mass ultrasound screening in Dangxiong County of Lhasa Prefecture and Dingqing County of Changdu Prefecture (eastern TAR).

## Materials and methods

### Study sites

**Fig. 1:**
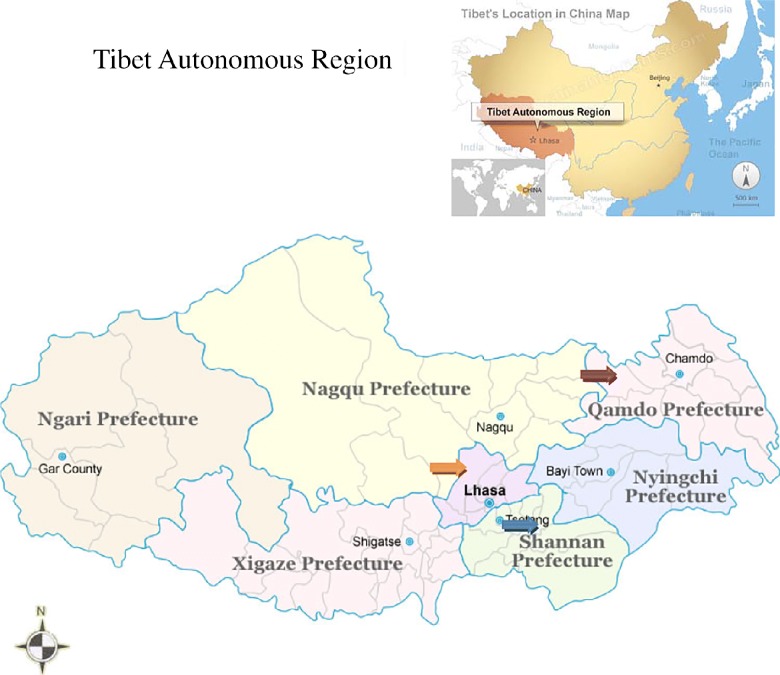
The three counties in Tibet surveyed for human echinococcosis, using historical data from Qusong (blue arrow) in 2003 and ultrasound screening of communities in Dangxiong (orange arrow) in 2006 and Dingqing (red arrow) in 2007.

In 2006 and 2007 two communities (one each in Dangxiong and Dingqing counties) in TAR (fig. 1) were investigated by mass screening for abdominal echinococcosis, and in addition hospital records in the People's Hospital of Changdu Prefecture were reviewed for human echinococcosis cases.

Dangxiong County is situated in the north of Lhasa Prefecture, adjoining Naqu Prefecture. Human CE cases have previously been reported from the county (Gong *et al.*, 2001). Dangxiong County covers an area of 10,036 km^2^, with an average elevation of 4200 m and a population of 42,000 (in 2006). The population comprised 98.8% Tibetans living in six pastoral communes and two townships. Domestic animals included goats, sheep, yaks and horses. The average annual temperature is 1.3°C (mean range − 10.4 to 10.7°C, greatest range − 32.5 to 26.5°C). Wild ungulates in Dangxiong County included Tibetan wild ass (*Equus kiang*), blue sheep (*Pseudois nayaur*) and yellow sheep (*Procapra picticaudata*). Common small mammals in Dangxiong County included plateau pika (*Ochotona curzoniae*), Himalayan marmot (*Marmota himalayana*), Tibetan hare (*Lepus oiostolus*) and voles (*Microtus* spp.).

Dingqing County (Tibetan: *Dengqen* or *Temgchen*), is one of 11 counties in Changdu Prefecture (also called *Chamdo* or *Qamdo*) located in the north-east of TAR, and adjoins Qinghai Province to the north and Sichuan and Yunnan provinces to the east. The average elevation of Dingqing County is 4000 m, with an area of 11,365 km^2^ that includes mostly pasture with just 110,000 ha of agricultural land and 6.7% forest. The average annual rainfall is 641 mm and the average annual temperature 3.1°C (full range − 25 to 27°C). The population was approximately 62,500 of which >99% were of Tibetan ethnicity. Most residents (99%) worked as herdsmen or farmers in two townships and 65 villages of Dingqing County. Livestock in north-west Dingqing County were yak, cattle, yak–cattle cross-breeds, sheep, goats, horses, donkeys and mules. The main agriculture products were barley, wheat and peas from south-east Dingqing. In addition, about 60% of the population was involved with the annual spring collection from pasture topsoil of ‘winter worm’ or *yartsa gunbu*, a caterpillar fungus (*Cordyceps sinensis*) that is a highly valued traditional Chinese medicine. Domestic dogs (owned and stray) were very numerous around towns or villages, and there were approximately 1–3 dogs per household. Common wild mammals in the county included Tibetan fox (*Vulpes ferrilata*), red fox (*Vulpes vulpes*), wolf (*Canis lupus*), plateau pika (*O. curzoniae*), microtine voles (*Microtus* spp.), Himalayan marmots (*M. himalayana*) and Tibetan woolly hares (*L. oiostolus*).

### Human screening

Community surveys for human echinococcosis were undertaken based upon voluntary self-selection, and included a questionnaire, registration, ultrasound scanning and rapid serological testing using dot immunogold filtration assay (DIGFA) (Feng *et al.*, 2010). All screened people agreed voluntarily to attend the survey and gave informed consent. The registration questionnaires included general information – name, ethnicity, sex, age, occupation, education level, annual income, etc. – and were performed by local Tibetan-speaking Centre for Disease Control (CDC) staff for exact records.

In Dangxiong County, two communes (Wuma and Ningzhong) and one town (Yangbajin) were screened in October 2006 (total population approximately 18,000). In total, 557 persons volunteered for screening, including 212 males and 345 females, 532 persons originated from the above areas and 25 from other communes (excluding Namco). In total, 488 people volunteered to donate 2–3 ml venous blood for rapid serological test by DIGFA, and among those 165 were tested further by standard enzyme-linked immunosorbent assay (ELISA) at Xinjiang Medical University Hospital (Urumqi) according to Feng *et al.* (2010). In Dingqing County, 232 people from three communes/towns (total population approximately 18,500) were screened in 2007. In total 195/232 persons donated 2–3 ml venous blood for DIGFA sero-testing.

The volunteers were given an abdominal ultrasound scan (portable ultrasound GE LOGO XP, China) by experienced sonographers (L. Yang, B. Bartholomot). Ultrasound hepatic images were recorded as CE, AE, query, other lesions or normal. Those who had characteristic CE or AE ultrasound images or a query lesion were recorded. All CE or AE cases confirmed in the survey were offered a free 6-month course of albendazole treatment. Cases of CE diagnosed in Dangxiong were treated surgically (cystectomy) in the People's Hospital of Lhasa City by surgeons from Xinjiang Medical University Hospital with the cooperation of surgeons from the People's Hospital of Lhasa City.

### Serology

A rapid DIGFA developed by Feng *et al.* (2010), available in kits (from Xinjiang Bestmind Biological Technique Development Ltd, Urumqi, China), was performed within 1 or 2 h after ultrasound scan. Blood samples were centrifuged at 2000 *g* after 1 h (if using general blood collection tubes) or 10 min (if using procoagulant tubes) and sera were tested immediately. DIGFA used four native antigen preparations: crude *E. granulosus* cyst fluid antigen (EgCF), *E. granulosus* protoscoleces extract (EgP), *E. granulosus* cyst fluid antigen B (AgB) and *E. multilocularis* laminated layer antigen (Em2) (Feng *et al.*, 2010). If either one of the EgCF, EgP, AgB or Em2 spots appeared (red), the sera were determined to be positive for echinococcosis; otherwise, if none appeared, the sera were considered negative. Positive, query or negative spots were recorded for the four antigens respectively. Positives with any one of four antigens were recognized as sero-positive. Results positive with AgB and negative or weakly positive with Em2 (e.g. antigen B ‘+’ and Em2 ‘–’ or ‘ ± ’) were indicative of antibodies to CE. Positives against Em2 and negative or weakly positive with AgB (e.g. Em2 ‘++’ and antigen B ‘ − ’, ‘ ± ’ or ‘+’) were recognized as indicative of the presence of antibodies to AE (Feng *et al.*, 2010).

In addition to serum samples collected in association with the active ultrasound-based screening in Dangxiong and Dingqing counties, a panel of archived sera was available from an earlier ultrasound survey (2003, *N*= 722) carried out in Qusong County (Shannan Prefecture) in south-central TAR (fig. 1). Archived sera were tested by DIGFA and matched to respective ultrasound results, but without personal details and questionnaire information. Serological positives and ultrasound-confirmed CE or AE cases were recorded and followed up as above.

### Review of published CE and AE reports for TAR (1980–2007)

Scientific papers on echinococcosis in Tibet Autonomous Region were identified in a Chinese academic search engine (CNKI, Wanfang and VIP) for the Chinese publications, as well as in the Medline/Pubmed search engine for international publications.

### Data analysis

Data from questionnaires, ultrasound and serological results were analysed using SPSS 16.0 (SPSS Inc., Chicago, Illinois, USA).

## Results

A search of published studies from Chinese and international literature (1980–2007) relating to human echinococcosis in the TAR identified 27 publications, of which 15 were most relevant.

Reports confirmed that human CE (total cases *n*= 1641) has been recorded in all seven prefectures in the TAR (table 1). In contrast, human AE cases (*n*= 22) were recorded only in Lhasa and Naqu prefectures and two eastern regions (Changdu and Linzhi prefectures) of TAR (table 1). One of the earliest reports of echinococcosis in the People's Hospital of Tibet AR (in Lhasa city) included 174 CE cases and one AE case for the period 1960–1983 but without details of case origins (Hu *et al.*, 1987). In the past 20 years, most human CE cases were recorded in prefectural hospitals in Naqu (*n*= 623), Shannan (*n*= 268) and Changdu (*n*= 182). For Lhasa, 174 CE and 162 CE cases were reported during the period 1960–1988 from the two main public hospitals and, in addition, 80 CE cases from a military hospital in Lhasa (Hu *et al.*, 1987; Ciren & Wang, 2000; Gong *et al.*, 2001; Zhou *et al.*, 2002; Liao *et al.*, 2003; Peng *et al.*, 2004; Yu, 2006; Cai *et al.*, 2007). Human CE cases were also reported in Rikaze, Linzhi and Ali prefectures in western TAR, but numbers were small (seven hepatic and two uterine CE cases in Rikaze, and 18 hepatic CE cases with computed tomography (CT) scan confirmation in Linzhi Prefecture Hospital) (Hou *et al.*, 2005; Duan *et al.*, 2006) (table 1).

**Table 1:** Human cystic (CE) and alveolar (AE) echinococcosis recorded from hospital cases in the Tibet Autonomous Region, P.R. China from 1960 to 2007; *N*= number of cases examined.

CE	AE
Prefecture	Period	*N*	References	Prefecture	Period	*N*	References
Naqu	1996–2000	623	Gong *et al.*, 2001	LhasaNaquShannan Changdu					1960–1992	12	Luo & Peng, 1993
Lhasa	1996–2000	35	Gong *et al.*, 2001			
Changdu		94	Luo & Zhao, 1994			
	1988–1995	70	Lan *et al.*, 1999			
	1996–2000	10	Gong *et al.*, 2001			
		68	Yin & Wang, 2007			
Shannan	1996–2000	22	Gong *et al.*, 2001	Changdu	1977	2	Peng, 1988
	1995–2001	80	Zhou *et al.*, 2002		2006	4	Hospital CT images
	1995–2004	116	Peng *et al.*, 2004				
	1988–2002	268	Liao *et al.*, 2003				
	1999–2006	59	Yu, 2006				
		155	Cai *et al.*, 2007				
Linzhi	1996–2000	8	Gong *et al.*, 2001	Linzhi	2006	3	Duan *et al.*, 2006
		18	Duan *et al.*, 2006				
Rikaze	1996–2000	4	Gong *et al.*, 2001				
		9	Hou *et al.*, 2005				
Ali	1996–2000	2	Gong *et al.*, 2001	Sichuan origin	2001	1	Yixi *et al*, 2001
Total		1641				22	

Reports of human AE cases have been sporadic in TAR, in contrast to Tibetan communities in neighbouring Sichuan and Qinghai provinces to the east and north-east of TAR but still situated on the Tibetan Plateau (Li *et al.*, 2005, 2010). After a first reported AE case in 1987, other reports showed two cases from Changdu (Peng, 1988), 12 cases from Lhasa, Naqu and Changdu prefectures (Yixi, 1992; Luo & Zhao, 1994), and three AE cases diagnosed by CT scanning in Linzhi (Duan *et al.*, 2006) (table 1).

We undertook a retrospective search of local patient records at the Changdu Prefecture Hospital for the period 2001–2005 and identified 61 CE cases and four putative AE cases. From those records 17 CE and three AE cases were confirmed to reside in Dingqing County in eastern TAR.

Previous mass ultrasound screenings in communities have also detected 48 CE cases in Lhasa Prefecture and nine CE cases in Qusong County of Shannan Prefecture (Hu *et al.*, 1999; unpublished study in 2003). No AE cases were specifically identified in these previous community studies in TAR. In the current study, pilot ultrasound screening (combined with rapid serology) was undertaken in Dangxiong County (Lhasa Prefecture) and Dingqing County (Changdu Prefecture). Data were also available from an earlier mass screening programme in Qusong County (table 2). The prevalence of human CE by ultrasound was 9.9% (55/557) in Dangxiong, 4.7% (11/232) in Dingqing and 1.2% (9/722) in Qusong (table 2). Human AE cases were only confirmed in the Dingqing survey but with an apparently high prevalence of 5.2% (12/232) including one mixed AE/CE case (table 2). Two query AE cases were, however, also listed from the Qusong survey. Sero-testing gave sero-prevalences >6% in Qusong and Dangxiong, but a significantly higher sero-prevalence (35.4%) for the Dingqing survey (chi-square *P* <  0.001) (table 2).

**Table 2:** The prevalence (%) of human cystic (CE) and alveolar (AE) echinococcosis screened by ultrasound or serology in Qusong, Dangxiong and Dingqing counties, TAR from 2003 to 2007; *N*=number of cases examined.

Counties	*N*	CE (%)	AE (%)	Mixed CE/AE (%)	Sero-positivity
*N*	Positives (%)
Qusong	722	9 (1.2)	0	0	722	55 (7.6)
Dangxiong	557	55 (9.9)	0	0	488	31 (6.4)
Dingqing	232	11 (4.7)	11 (4.7)	1 (0.4)	195	69 (35.4)
Total	1511	75 (4.96)	11	1	1405	155 (11.0)

The incidence of human CE was estimated from these hospital records for the seven prefectures of TAR, and ranged from 1.9 to 155 per 100,000, with a mean incidence throughout TAR of 45.1 per 100,000 (table 3). Human AE incidence was based on smaller datasets but was estimated to range between 0.6 and 2.8 per 100,000, with the highest incidence in Changdu Prefecture in eastern TAR.

**Table 3:** Estimated incidences (per 100,000) and confirmed cases of human cystic (CE) and alveolar (AE) echinococcosis in TAR, relative to prefecture from 1960 to 2005.

Prefecture	Population(2005)	CE	AE
Confirmed	Incidence	Confirmed	Incidence
Lhasa	540,500	138	25.5	3	0.6
Naqu	401,871	623	155.0		
Ali	86,277	2	2.3		
Rikaze	662,146	13	1.96		
Linzhi	158,167	26	16.4	3	1.9
Shannan	325,063	268	82.4		
Changdu	606,444	182	30.0	17	2.8
Tibet AR	2,778,463	1252	45.1	23	0.8

## Discussion

Previous studies have identified very high prevalences of both CE and AE in Tibetan communities in western Sichuan and south-eastern Qinghai provinces of China (outside the TAR), with mean prevalences of up to 15% at the township level for both CE and AE. These prevalences are among the highest in the world. However, despite having a considerable Tibetan population, there is little data about CE, and especially AE, in the TAR itself.

Hospital records in TAR listed 709 cases of human CE from three hospitals in Naqu Prefecture for the period 1996–2000 (Gong *et al.*, 2001). In that study 623 CE cases were originally from Naqu Prefecture and the other CE cases were from the prefectures of Lhasa (*n*= 35), Shannan (*n*= 22), Changdu (*n*= 10), Linzhi (*n*= 8), Rikaze (*n*= 4) and Ali (*n*= 2), and other areas (*n*= 5) (Gong *et al.*, 2001). A total of 94 cases of echinococcosis (not differentiated as CE or AE), were reported in Changdu Prefecture (Luo & Zhao, 1994). In Shannan Prefecture 116 (period 1995–2004), 155 (to 2007) and 268 (1988–2002) echinococcosis cases were reported (Liao *et al.*, 2003; Peng *et al.*, 2004; Cai *et al.*, 2007).

Most of the mass screening community studies in TAR to date have used the relatively non-specific Casoni skin test (the prevalence based on this test was estimated as 34.9% over the period 1988–1991 (Guo & Yu, 1994)). One survey in Dangxiong and Mozugongka counties of Lhasa Prefecture, confirmed 48 CE cases from 734 individuals testing positive for the Casoni skin test in a test population of 20,160 (Hu *et al.*, 1999). Mass screening using portable ultrasound scanning for abdominal CE (or AE) is a much more specific approach (Bartholomot *et al.*, 2002; Macpherson *et al.*, 2003; Li *et al.*, 2005).

Overall, human AE is a rare disease at the national level in China; however, significant foci of AE have been described in central, north-west and south-west China (Craig *et al.*, 1992; Zhou *et al.*, 2000; Li *et al.*, 2010). However, an important question remains: What is the extent of human AE within the TAR? This is pertinent especially because of the high prevalences of human AE detected in Tibetan communities on the eastern Tibetan plateau (but outside the TAR) in Sichuan and Qinghai provinces (Schantz *et al.*, 2003; Li *et al.*, 2010; Giraudoux *et al.*, 2013b). The first human AE case reported in TAR was in 1987 in the TAR People's Hospital, Lhasa (Hu *et al.*, 1987). In 1988 two AE cases (history identified from 1977) were reported from the eastern TAR in Changdu Prefecture (Peng, 1988). Since then, it appears that 12 AE cases were confirmed in hospitals in Lhasa, although their origins not only included cases from Lhasa Prefecture, but also from Naqu and Changdu prefectures (Pu, 1999; Yixi *et al.*, 2001). Later, a further three AE cases, confirmed by CT scanning, were reported in Linzhi Prefecture (Duan *et al.*, 2006). However, no AE cases were previously identified during mass screening community studies in TAR prior to the current study.

The objectives of the current study not only included a review of published literature relating to CE/AE in TAR, and also of patient records in the People's Hospital of Changdu Prefecture (TAR), but in addition to undertake pilot community studies in two counties: Dangxiong in Lhasa Prefecture, central TAR, and Dingqing in Changdu Prefecture, situated in eastern TAR. Community surveys included questionnaire registration, abdominal ultrasound scanning and DIGFA sero-testing. In addition, ultrasound records from an unpublished community study carried out in 2003 in Qusong County (Shannan Prefecture) were examined in detail. In these three pilot ultrasound-based community mass screenings, AE cases were only detected in Dingqing County (Changdu Prefecture) but with a high prevalence of 5.2% in the screened population (including a case of mixed AE/CE infection). No AE cases were identified in the 1279 people screened in Dangxiong and Qusong counties. In contrast, human CE cases have been found in all counties where mass screening was performed and have been treated in all seven prefectures of TAR (Gong *et al.*, 2001), with most hospital cases in Naqu, Shannan, Changdu, Lhasa and Linzhi prefectures. This supports previous community studies by the Chinese Ministry of Health (Ministry of Health, 2005), which found that human CE cases were located mainly in the south-east, east and north-central areas of TAR (table 3). Previous reports showed that the highest ultrasound prevalence for human CE in China occurred in Tibetan pastoral communities of the eastern Tibetan plateau, including south-west Qinghai and north-west Sichuan provinces (Qiu & Wang, 1999; Schantz *et al.*, 2003; Craig, 2004; Li *et al.*, 2010; Giraudoux *et al.*, 2013b). In the current pilot study in TAR we determined the point prevalence of ultrasound-diagnosed human CE as 9.9% (55/557) in Dangxiong County, and 4.7% (11/232) in Dingqing County; in addition, a review of ultrasound records from a prior screening in 2003 in Qusong County provided a CE prevalence of 1.2% (9/722). The prevalence of CE was significantly higher for Dangxiong (*P* <  0.01). Human CE incidence for the whole of TAR was estimated to be 45.1 per 100,000, with a range of 1.9 to 155 per 100,000 and the highest incidence in Naqu Prefecture. However, it is important to consider the limitations in these estimates, partly as a result of the challenges in estimating the incidence of an infection with such a long latent period before clinical signs manifest, and partly due to the biases associated with the use of hospital records rather than statistically sound surveys.

Transmission of *E. granulosus* in TAR is almost certainly related to the widespread distribution and high density of both dogs and domestic livestock (sheep, goats, yak) (Eckert *et al.*, 2001; Craig, 2004), especially in central and eastern areas (Giraudoux *et al.*, 2013a). *Echinococcus granulosus* was recorded from the intestines of dogs in Naqu (TAR), and was reported to be common in yak, cattle, sheep and goats in TAR (Zhang *et al.*, 1994; Nima, 1998; Lan *et al.*, 1999). The transmission ecology of *E. multilocularis* in TAR is not known, but will probably be similar to that described in Tibetan autonomous regions in north-west Sichuan and south Qinghai, i.e. a wildlife cycle involving Tibetan foxes (*V. ferrilata*) and a number of microtine and/or ochotonid small mammal species (Raoul *et al.*, 2006). Furthermore, the role of domestic dogs in the transmission of human AE in Tibetan communities in TAR is likely to be similar to that described in Tibetan communities in north-west Sichuan (Vaniscotte *et al.*, 2011; Giraudoux *et al.*, 2013a; Moss *et al.*, 2013; Wang *et al.*, 2015). The distribution of human AE disease in western China was found to be related to the proportion of alpine meadows/grasslands in a given region, which provide optimal habitats for potential small mammal intermediate hosts (Giraudoux *et al.*, 2013a). Co-occurrence of both CE and AE in the same patient, as identified in Dingqing County, would support the role of dogs in transmission. Such double infection has been identified at least twice in other regions of China (Ningxia and Xinjiang) (Wen *et al.*, 1992; Yang *et al.*, 2006), and double intestinal infection of a dog with *E. granulosus* and *E. multilocularis* has been demonstrated using molecular tools (Zhang *et al.*, 2006). Furthermore the ‘landscape risk model’ of Giraudoux *et al.* (2013b) predicted two human AE transmission hotspots in eastern TAR. One was in Dingqing, which appears to be confirmed by the current study, the other was to the north-west of Naqu, close to the border with Qinghai Province; however, to date no community screening has been reported from the latter area.

In summary, both human cystic echinococcosis (CE) and alveolar echinococcosis (AE) are endemic in the Tibet Autonomous Region (TAR). Human CE is relatively widespread, occurring in all of the seven prefectures of TAR, but with higher case numbers, incidence and prevalence in central and eastern areas of TAR. CE incidence was estimated to be 45.1 per 100,000 for the TAR. In contrast, fewer than 20 human AE cases have been reported from hospitals in central and eastern prefectures; however, a pilot mass screening using ultrasound revealed a significant AE prevalence (5.2%) in Dingqing County in the eastern prefecture of Changdu. It is anticipated that extended mass screening will detect more cases of human AE in the eastern region of TAR.
